# The role of TiO_2_ and gC_3_N_4_ bimetallic catalysts in boosting antibiotic resistance gene removal through photocatalyst assisted peroxone process

**DOI:** 10.1038/s41598-024-74147-4

**Published:** 2024-10-02

**Authors:** Xiaoyu Cong, Paweł Mazierski, Magdalena Miodyńska, Adriana Zaleska-Medynska, Harald Horn, Thomas Schwartz, Marta Gmurek

**Affiliations:** 1 Institute of Functional Interfaces (IFG), Microbiology/Molecular Biology Department, Karlsruhe Institute of Technology (KIT), Hermann von Helmholtz Platz 1, 76344 Eggenstein-Leopoldshafen, Germany; 2 Department of Molecular Engineering, Faculty of Process and Environmental Engineering, Lodz University of Technology, Lodz, 93-005 Poland; 3 Water Chemistry and Water Technology, Karlsruhe Institute of Technology, Engler-Bunte-Institut, 76131 Karlsruhe, Germany; 4 Department of Environmental Technology, Faculty of Chemistry, University of Gdansk, Gdansk, 80-308 Poland; 5 Water Chemistry and Water Technology, DVGW German Technical and Scientific Association for Gas and Water Research Laboratories, 76131 Karlsruhe, Germany

**Keywords:** Photocatalytic ozonation, Photocatalyst-assisted peroxone process, Bimetallic catalysts, Antibiotic-resistant genes, Regrowth, Antibiotic-resistant bacteria, Engineering, Chemical engineering, Civil engineering, Environmental sciences, Environmental chemistry, Environmental impact

## Abstract

Antibiotics are extensively used in human medicine, aquaculture, and animal husbandry, leading to the release of antimicrobial resistance into the environment. This contributes to the rapid spread of antibiotic-resistant genes (ARGs), posing a significant threat to human health and aquatic ecosystems. Conventional wastewater treatment methods often fail to eliminate ARGs, prompting the adoption of advanced oxidation processes (AOPs) to address this growing risk. The study investigates the efficacy of visible light-driven photocatalytic systems utilizing two catalyst types (TiO_2_-Pd/Cu and g-C_3_N_4_-Pd/Cu), with a particular emphasis on their effectiveness in eliminating *bla*_*TEM*_, *ermB*,* qnrS*,* tetM*. *intl1*, 16 S rDNA and 23 S rDNA through photocatalytic ozonation and peroxone processes. Incorporating O_3_ into photocatalytic processes significantly enhances target removal efficiency, with the photocatalyst-assisted peroxone process emerging as the most effective AOP. The reemergence of targeted contaminants following treatment highlights the pivotal importance of AOPs and the meticulous selection of catalysts in ensuring sustained treatment efficacy. Furthermore, Polymerase Chain Reaction-Denaturing Gradient Gel Electrophoresis (PCR-DGGE) analysis reveals challenges in eradicating GC-rich bacteria with TiO_2_ and g-C_3_N_4_ processes, while slight differences in Cu/Pd loadings suggest g-C_3_N_4_-based ozonation improved antibacterial effectiveness. Terminal Restriction Fragment Length Polymorphism analysis highlights the efficacy of the photocatalyst-assisted peroxone process in treating diverse samples.

**Supplementary Information:**

The online version contains supplementary material available at 10.1038/s41598-024-74147-4.

## Introduction

Antibiotics, a cornerstone of modern medicine, have revolutionized healthcare and saved countless lives^1^. However, their widespread use and improper disposal have led to their release into aquatic ecosystems, fueling the rapid emergence of antimicrobial resistance (AMR)^2,3^. The resulting increase in antibiotic-resistant bacteria (ARB) and antibiotic-resistant genes (ARGs) poses a global healthcare crisis, escalating mortality rates^4^. The abundance of ARB and ARGs is influenced by various factors, including water quality, seasonal variations, and the presence of pollutants like heavy metals and pesticides, which can enhance horizontal gene transfer (HGT)^5,6^. Additionally, intrinsic spontaneous mutations and heterologous expression contribute to antibiotic resistance^7^.

As highlighted, the propagation of antibiotic resistance is a swiftly expanding problem in aquatic habitats as a result of anthropogenic activities, veterinary care and farming^1,8^. Regrettably, even in extremely low antibiotic conditions, the evidence shows the acceleration of antibiotic resistance^9^. Numerous ARB and ARGs have been found not only in nosocomial wastewater and livestock farming wastewater, but also in surface water, effluent discharged from wastewater treatment plants (WWTPs)^10^, and even drinking water^11^. In particular, it is impossible to discover a place in surface water where AMR do not exist^12^; moreover, AMR has been identified in ground water at depths of up to 10 m^13^. Several studies have found that WWTPs and hospital wastewater provide ideal living conditions for most autochthonous bacteria carrying ARGs, and clinically relevant gram-negative pathogens and ARGs are found scuttling between various environments via WWTP effluent^14^. Conventional wastewater treatment fails to fully eliminate ARGs and ARB, highlighting the need for effective disinfection strategies in WWTPs. Unlike traditional methods, Advanced Oxidation Processes (AOPs) use oxidants like ozone and hydrogen peroxide to degrade contaminants in water^15^. The mechanisms of AOPs involve the generation of highly reactive species like hydroxyl radicals (^●^OH), which are powerful oxidants capable of breaking down ARB and ARGs^16^. However, understanding bacterial regrowth post-disinfection, especially involving ARGs, is crucial for ecological balance^17^. The reactivation of bacteria into a viable but non-culturable (VBNC) triggered by extreme environmental stressors such as UV radiation and oxidative stress, highlights the importance of implementing appropriate treatment strategies to prevent regrowth in downstream systems^18,19^.

Heterogeneous catalytic ozonation (HCO) provides a viable disinfection strategy by using catalysts to boost the decomposition of ARB and ARGs. The careful selection of catalysts and the optimization of reaction parameters in HCO operations are vital for effective disinfection and the reduction of ARB and ARGs in water treatment endeavors^20^. Photocatalytic disinfection was proposed after successful bacteria inactivation using semiconducting materials like TiO_2_, SnO_2_, Fe_2_O_3_, ZnO, and g-C_3_N_4_^21–23^. Commercial P25 Degussa, comprising anatase and rutile crystallinity, demonstrates enhanced pollutant inactivation under UV light, necessitating modification for visible light utilization through metal-ion doping or bimetallic catalysts^24,25^. Rutile TiO_2_ offers superior photocatalytic potential due to its stability and extended light response range, yet anatase TiO_2_ often exceeds it in efficiency^26^. Doping of rutile catalysts, as demonstrated by Gmurek et al.2023^25^, addresses this limitation, showing higher efficiency in degrading ARGs compared to P25 TiO_2_. Graphitic carbon nitride (g-C_3_N_4_), primarily composed of carbon and nitrogen, emerges as a notable and sustainable photocatalyst, offering adaptability and ease of doping for enhanced efficiency in various applications^27–29^. Selected alongside TiO_2_ for its low solubility, high-surface area, and tunable band gap, g-C_3_N_4_ can be synthesized in various morphologies, offering versatility in contaminant removal^30,31^. Metal dopants play a crucial role in narrowing band gaps and generating highly oxidized species, making metallic nanoparticles essential for harvesting visible light through surface plasmon resonance^32^.

The discovery of synergistic effects, including enhanced ^●^OH generation from combining treatment strategies, has spurred exploration into introducing additional oxidant species like hydrogen peroxide or ozone to improve photocatalytic processes^33^. Photocatalytic ozonation has shown oxidation rates 2.3 to 4.4 times higher than photocatalytic processes alone, demonstrating the potential of integrated AOPs for wastewater treatment^33^. The synergistic effects of advanced oxidation and photocatalysis have been extensively studied in various combinations by Demir et al., 2019^34^. Key observations include: (1) the application of UV light significantly enhances hydrogen production, and (2) the highest chemical oxygen demand (COD) degradation rate, achieving 97.9%, is observed when advanced oxidation is combined with photocatalysis. This combination is critical for optimizing the efficacy of water treatment processes. Additionally, the synergistic effects of AOPs are known to target and degrade critical organic components within microorganisms, thereby causing significant damage to ARB and ARGs^35^. The combination of ozone and hydrogen peroxide, known as the peroxone process, accelerates the decomposition of ozone, leading to the generation of a higher reactive oxygen species (ROS). The underlying reaction mechanisms of the peroxone process can be illustrated through the following equations, which demonstrate how ozone reacts with the deprotonated form of hydrogen peroxide, ultimately resulting in the production of increased amounts of ROS^36,37^ (Eqs. 1–7):

1 $${{\text{H}}_{\text{2}}}{{\text{O}}_{\text{2}}} \to {\text{H}}{{\text{O}}_{\text{2}}}^{ - }+{{\text{H}}^+}$$

2 $${\text{H}}{{\text{O}}_{\text{2}}}^{ - }+{{\text{O}}_{\text{3}}} \to {\text{H}}{{\text{O}}_{\text{2}}}^{ \cdot }+{{\text{O}}_{\text{3}}}^{{ \cdot - }}$$

3 $${\text{H}}{{\text{O}}_{\text{2}}}^{\cdot} \to {\text{H}}+{{\text{O}}_{\text{2}}}^{{\cdot - }}$$

4 $${\text{H}}{{\text{O}}_{\text{2}}}^{\cdot}/{{\text{O}}_{\text{2}}}^{{\cdot - }}+{{\text{O}}_{\text{3}}} \to {{\text{O}}_{\text{2}}}+{{\text{O}}_{\text{3}}}^{{\cdot - }}$$

5 $${{\text{O}}_{\text{3}}}^{{\cdot - }}+{{\text{H}}^+} \to {\text{H}}{{\text{O}}_{\text{3}}}^{\cdot}$$

6 $${\text{H}}{{\text{O}}_{\text{3}}}^{\cdot}/{{\text{O}}_{\text{3}}}^{{\cdot - }} \to {\text{H}}{{\text{O}}^\cdot}+{{\text{O}}_{\text{2}}}$$

7 $${\text{H}}{{\text{O}}^\cdot}+{{\text{H}}_{\text{2}}}{{\text{O}}_{\text{2}}}/{\text{H}}{{\text{O}}_{\text{2}}}^{ - } \to {{\text{H}}_{\text{2}}}{\text{O}}+{{\text{O}}_{\text{2}}}^{{\cdot - }}$$

While, the Photocatalyst-Assisted Peroxone Process is a highly advanced oxidation system that integrates the photocatalytic properties of photocatalysts with the chemical interactions of the peroxone process (H₂O₂/O₃), resulting in enhanced production of ROS for the degradation of organic pollutants^36,38,39^. During photocatalytic reactions, the photocatalyst is irradiated with enough energy to generate electron-hole pairs. The photogenerated holes on the photocatalyst surface are effective oxidizing agents that interact with water or hydroxide ions, resulting in the generation of potent hydroxyl radicals (HO•), while the e^−^ react with the dissolved oxygen to produce superoxide anions (O₂•⁻)^38,39^. Simultaneously, the conduction band electrons reduce external oxidants like hydrogen peroxide (H₂O₂) and ozone (O₃), producing additional ROS such as O₂•⁻ and further hydroxyl radicals^38,39^. The peroxone process plays a critical role by accelerating the decomposition of ozone through its reaction with hydrogen peroxide, resulting in a cascade of reactions that generate a variety of ROS. The synergy between the photocatalytic activity and the peroxone process significantly amplifies the overall oxidative potential of the system, facilitating the breakdown of recalcitrant organic compounds. The integration of photocatalysis with the peroxone process exemplifies a robust strategy for AOPs, offering a powerful tool for environmental remediation and wastewater treatment applications.

Although photocatalytic disinfection processes are known to reduce the abundance of antibiotic ARGs, they often fall short of effectively preventing the spread of antibiotic resistance due to insufficient generation of ROS. This inadequacy affects the ability to fully disrupt DNA and prevent the conjugative transfer of ARGs in aquatic environments, regardless of the light or catalysts used^16,40^. Additionally, the application of photocatalyst-assisted peroxone processes for the removal of ARB and ARGs has not yet been investigated, presenting a promising avenue for future research. This study highlights the novel aspect of applying the photocatalyst-assisted peroxone process to address this gap. The integration of an electron acceptors, such as H₂O₂ and O₃, in combination with the photocatalyst, is proposed as a promising approach to enhance ROS generation and improve the effectiveness of ARG removal. As a consequence, the nanoscale catalysts TiO_2_-rutile and g-C_3_N_4_ pure and after bimetallic modification by Cu and Pd have been studied. The main goal of the study was to apply visible light as a driving force for photocatalytic disinfection. To increase the efficiency, apart from TiO_2_-rutile and g-C_3_N_4_-pure, modified catalysts with different Cu and Pd loadings (1%, 05%, 01%) wre also explored for AMR removal. The study investigates the efficacy of visible light-driven photocatalytic systems utilizing two catalyst types (TiO_2_-Pd/Cu and g-C_3_N_4_-Pd/Cu), with a particular emphasis on their effectiveness in eliminating ARGs (*bla*_*TEM*_, *ermB*,* qnrS*,* tetM)*,* intl1 (*a specific marker for class 1 integrons ), 16 S rDNA (eubacterial ribosomal gene marker) and enterococci (taxonomic gene marker, 23 S rDNA) through photocatalytic ozonation and peroxone processes. Additionally, this study aims to elucidate the varying abilities of the AOPs to prevent the post-treatment reemergence of investigated targets during dark storage, considering the influence of the catalysts applied. A parallel analysis of biodiversity changes complements the investigation, providing a holistic understanding of the treatment’s effectiveness against all targeted genetic markers.

## Materials and methods

### Sample collection and processing

The secondary effluent (labelled on all graphs as “Sec. effluent”) were freshly collected from investigated WWTP (Karlsruhe Neureut Wastewater Treatment Plant), which is located in the south-west of Germany and treats an average of 93,000 m^3^d^− 1^ of WW each day representing 875,000 population equivalents. Sampling campaigns were performed between January 2022 and March 2022, the water samples were then collected in sterile plastic bottles, sent refrigerated to the lab, and examined within 24 h. Water samples were collected in one consecutive week, and the character and parameter values are shown in the following Table S1. Given the experimental time constraint of a maximum duration of six months, the number of samples subjected to each variant of the AOPs treatment was limited to a maximum of two or three (*n* = 2/3).

To obtain a sufficient quantity of biomass for DNA extraction both pre- and post-application of AOPs, the secondary effluent was subjected to vacuum filtration utilizing polycarbonate membranes with a pore size of 0.2 μm (Whatman Nuclepore Track-Etched Membranes, Sigma-Aldrich, Munich, Germany). Therefore, intact ARB carrying ARGs can be intercepted, followed by DNA extraction for further analysis. However, the specific types of ARB present in the samples were not confirmed in this study. Notably, only one facultative pathogenic bacteria (FPB), enterococci, was detected. Since the filtration volume depends on the turbidity, the volumes of each sample were 100 mL for the effluent from WWTPs, 200 mL for the effluent after advanced treatment in this investigation. All filtered membranes were submerged in a solution using a 1.5 mL Eppendorf kit (SafeSeal tubes, Carl Roth, Karlsruhe, Germany) containing 400 µL of 25 µM propidium monoazide (PMA, Biotium, Hayward, California, USA). Following a 5-minute incubation period in the dark, PMA-treated samples were subjected to the PhAST Blue PhotoActivation System at 100% intensity for 15 min to enhance dye crosslinking with DNA. After the filtration process, the filters were carefully placed into Eppendorf tubes. These tubes were then stored at -20 °C to preserve the samples until they could be processed further. The graphical data depicted was obtained exclusively from the samples subjected to propidium monoazide (PMA) treatment. The analysis focused exclusively on the intracellular DNA present in viable bacterial cells, thereby providing a more accurate representation of the actual bacterial population.

### Preparation of photocatalysts

#### Synthesis of pristine TiO2 and g-C3N4 materials

Pristine rutile TiO_2_ exhibiting urchin-like morphology was obtained according to the procedure described in our previous work^41^. Concentrated hydrochloric acid was introduced into the Teflon vessel in the amount of 60 mL and then 68 g of titanium (IV) n-butoxide TBOT (68 g) was added to it. Above mixture was stirred for 10 min and then heated at 170 °C for 24 h in Teflon-lined stainless-steel autoclave. Then the resulting white precipitate was cleaned several times by washing with water and ethanol and finally dried at 70 °C and calcined at 300 °C for 1 h. Thermal polymerization method was used to obtain g–C_3_N_4_ in accordance to procedure described in our previous paper^42^. In brief, 10 g of melamine were placed in a ceramic crucible with a cover. After closing the ceramic crucible, the synthesis was carried out in an oven at 550 °C for 4 h with a heating rate of 2 °C/min. Obtained powder was ground to a homogeneous form and cleaned several times with the use of methanol and water. In the last step, the yellow powder was dried at 60 °C for 12 h.

#### Synthesis of bimetallic Pd/Cu-TiO2 and Pd/Cu-g-C3N4

The photodeposition method was used to deposit bimetallic Pd/Cu on TiO_2_ and g-C_3_N_4_. The photodeposition was carried out in an 80 ml quartz glass photoreactor with a cooling jacket at a temperature of 10 °C. In the first step, the photocatalyst (TiO_2_ or g-C_3_N_4_) and 60 ml of ethanol were introduced into the photoreactor, and then at the same time the copper and palladium precursor (aqueous solution of potassium tetrachloropalladate(II) and copper diacetate monohydrate). The above mixture was allowed to stir in dark under nitrogen atmosphere for 30 min. The degassed and homogeneous mixture was irradiated with UV-Vis (Xenon lamp, Oriel, 1000 W) through 1 h. After photodeposition of bimetallic Pd/Cu, the suspension was cleaned several times with ethanol and dried at 60 °C for 12 h. The product was washed several times with water and ethanol and centrifuged at 6000 rpm. Finally, the product was dried at 60 °C for 12 h. The amount of deposited bimetallic Pd/Cu nanoparticles was changed by increasing the amount of palladium and copper precursor introduced into the photoreactor, and all the photocatalysts used in our investigation are documented in Table 1.

**Table 1:** Nomenclature and abbreviations for the photocatalysts examined in this work.

Photocatalyst with nominal metal content	Nomenclature
TiO_2_ rutile nanostructure	TiO_2_ (rutile)
TiO_2_ rutile nanostructure modified by 0.1% Cu/Pd	(Pd/Cu)^0.1%^TiO_2_
TiO_2_ rutile nanostructure modified by 0.5% Cu/Pd	(Pd/Cu)^0.5%^TiO_2_
TiO_2_ rutile nanostructure modified by 1% Cu/Pd	(Pd/Cu)^1%^ TiO_2_
g-C_3_N_4_ pure nanostructure	g-C_3_N_4_(pure)
g-C_3_N_4_ pure nanostructure modified by 0.1% Cu/Pd	(Pd/Cu)^0.1%^g-C_3_N_4_
g-C_3_N_4_ pure nanostructure modified by 0.5% Cu/Pd	(Pd/Cu)^0.5%^g-C_3_N_4_
g-C_3_N_4_ pure nanostructure modified by 1% Cu/Pd	(Pd/Cu)^1%^ g-C_3_N_4_

### Catalysts characterization

The obtained materials were characterized in terms of surface morphology by field-emission scanning electron microscopy (FE-SEM, JEOL JSM-7610 F) and optical properties by recording diffuse reflectance spectra on a UV–vis spectrophotometer (UV 2600, Shimadzu, BaSO_4_ was used as a reference). HAADF-STEM image with EDXS maps, XPS analysis, Raman spectra and pXRD patterns of bimetallic catalysts are reported in our previous paper^25^.

### Photocatalytic experiments

A 500 mL glass reactor, maintained under continuous magnetic stirring, served as the vessel for all reactions. Photo-assisted reactions utilized a Sol3A Solar Simulator (Newport) to simulate sunlight. Ozone, generated from a pure oxygen stream using a BMT 803 N ozone generator (BMT Messtechnik GMBH, Germany), was detected in each stream using inlet and outlet gas analyzers. Additionally, a potassium iodide (KI) solution trapped residual ozone in the outlet gas stream. Ozone was introduced into the inlet gas stream at a flow rate of 0.4 Lmin^-1^ and a concentration of 10 mgL^-1^ (resulting in a specific ozone dose of 1 g O_3_ g DOC^-1^ and a *k*_L_*a* value of 0.72 min^-1^). Metal-doped TiO_2_ (rutile) and g-C_3_N_4_ nanostructures were applied in photocatalytic investigations. In the study, the catalyst concentration was set to 150 mgL^-1^. Consequently, to prepare the mixture, 60 mg of the catalyst was incorporated into 400 mL of secondary effluent from the WWTP. The catalyst was introduced 15 min before the experiment started in case it would have a negative influence on the performance. The produced ozone was then introduced to a batch reactor containing 400 mL of the effluent samples, which were placed in the sunlight simulator’s light chamber. The catalysts had been properly mixed with the tested liquids, as evidenced by a gas diffuser producing small bubbles in the liquid samples. The appendix included a process flow schematic for the photocatalytic ozonation unit. The value of the inlet and outlet ozone meters was recorded during the experiment to examine the O_3_ equilibrium in the reactor. In addition to photocatalytic ozonation trials, the identical setup for photocatalysis was also used with additional H_2_O_2_ and without ozone (Fig. S1). In one of the ozone-driving studies, 400 mL of treated effluent was spiked with 42 µL of 30% H_2_O_2_ solution (0.92 mM of H_2_O_2_) to test if this would increase the removal efficiency of FPB and ARGs. Apart from that, all the photocatalytic trails required to be examined for bacterial regrowth after 1 h of treatment, 200 mL of treated secondary effluent was maintained in a dark cabinet at room temperature for 72 h. Table 2 compiles all process variables.

**Table 2:** Procedures performed regarding the trials, including the parameters of several methods (cat.: all catalysts listed in Table 1, visual sunlight simulator: Vis (1000 W), ozone inflow concentration: 37 g N^− 1^m^− 3^, H_2_O_2_ concentration: 0.92 mM of H_2_O_2_, catalyst concentration: 150 mg L^− 1^).

Experiments	Parameters	Working volume (mL)	Time (h)	Light
Ozonation	O_3_	400	1	−
Photocatalytic oxidation	Cat./Vis	400	1	+
Photocatalytic ozonation	Cat./Vis/O_3_	400	1	+
Photocatalyst-assisted peroxone process	Cat./Vis/O_3_/H_2_O_2_	400	1	+
Bacterial regrowth	Bacterial	200	24/72	−

### DNA extraction

DNA was extracted by using the FastDNA TM Spin Kit for Soil (MP Biomedicals, Santa Ana, USA) and FASTPREP^®^ instrument (MP Biomedicals, Santa Ana, USA). For mechanical cell disruption, the filtrated membranes were put in the Lysing Matrix E tube. Proteins were then separated by centrifugation and precipitation, and the DNA was finally purified by attaching to a silica matrix. The next steps in the DNA extraction process were carried out according to Hembach et al., 2019. The concentration of the extracted DNA was measured by NanoDrop (ND-1000, PEQLAB Biotechnologie GmbH, Germany) and the Quant-iTTM PicoGreen^®^ dsDNA Assay Kit (Thermo Fisher Scientific, Nidderau, Germany).

### qPCR analysis

On a Bio-Rad Cycle CFX96 (CFX96 TouchTM Deep Well Real-Time PCR Detection System, Bio-Rad, Munich, Germany), SYBR Green qPCR experiments were carried out in technical duplicates, and the analysis was done using the manufacturer’s software (Bio-Rad CFX Manager Software). Reactions were run in volumes of 20$\:{\upmu\:}\text{L}$, containing 10$\:{\upmu\:}\text{L}$ Maxima SYBR Green/ROX qPCR Master Mix (2$\:\times\:$) (Thermo Scientific Nidderau, Germany), 7$\:{\upmu\:}\text{L}$ nuclease-free water (Ambion, Life technologies, Karlsbad, Germany), 1$\:\:{\upmu\:}\text{L}$ of Forward Primer (FW) (5 $\:{\upmu\:}\text{M}$), 1 $\:{\upmu\:}\text{L}$ of reverse primer (Rev) (5$\:\:{\upmu\:}\text{M}$), and 1 $\:{\upmu\:}\text{L}$ of template DNA. Aside from that, no template control (NTC) was considered as a contamination control sample on the plate. The denaturation step uses high temperature (normally 95 °C) for 10 min to melt double-stranded DNA into single strands. Consequently, for primer annealing and extension, heated to 95 °C for 15 s before cooling to 60 °C lasting 1 min. To evaluate the specificity of the amplification, melting curves were recorded by rising the temperature from 60 °C to 95 °C (1 °C every 10 s). Table S2 (Appendix) provides the primer sequences, calibration line equation, efficiency values, detection limits, correlation coefficient value of the calibration lines, amplicon size, and references used for each target, including facultative pathogenic bacteria and specific antibiotic resistance genes.

### PCR-DGGE and t-RFLP

The amplification of eubacterial 16 S rDNA was conducted through Polymerase Chain Reaction (PCR) utilizing primers GC27F and 517R, as detailed in Table S2 provided in the Appendix. The working solution for each sample was prepared, comprising 19 µL of nuclease-free water sourced from Ambion, Life Technologies, Karlsbad, Germany, along with 2.5µL of buffer (15 mM MgCl_2_), 0.5µL of dNTP (10 mM), 0.125 µL of Polymerase (TEMPase Hot Start DNA Polymerase), 1 µL of GC27F, 1 µL of 517R, and 1 µL of the respective sample. Subsequently, the PCR samples were applied onto a vertically-oriented gel composed of acrylamide/bis-acrylamide with a urea gradient ranging from 40 to 70%. Following gel electrophoresis, the gels were treated with GelRed™ for staining and subsequently subjected to visualization of DNA band profiles by iBright™ CL1500 imaging system (Thermo Fisher Scientific, Waltham, United States).

T-RFLP is a molecular biology technique for analyzing microbial community diversity. It involves DNA extraction, PCR amplification of a target DNA region, digestion with restriction enzymes to produce labeled terminal 16 S rRNA fragments, and fragment size analysis to infer microbial community composition. Sample preparation includes creating technical duplicates for result reliability. The DNA-based t-RFLP analysis starts with universal PCR using a FAM-labeled primer system targeting the V1-V3 region of the 16 S rRNA gene, with primer sequences specified in Table S2.

The PCR reaction mix includes 19 µL of nuclease-free water, 2.5 µL of combination buffer, 0.5 µL of dNTPs sourced from VWR in Darmstadt, Germany, 0.125 µL of Hot Start Polymerase (also from VWR, Darmstadt, Germany), and 1 µL of each primer, culminating in a final volume of 25 µL for each reaction. The PCR cycling conditions are meticulously set to include an initial denaturation phase at 95 °C for 15 min, followed by 30 cycles of denaturation at 95 °C for 30 s, annealing at 56 °C for 30 s, and elongation at 72 °C for 60 s, with a final elongation step at 72 °C for 10 min. Following PCR amplification, the product undergoes restriction digestion with the Fast Digest Enzyme *HhaI* from (Thermo Fisher Scientific, Waltham, United States), employing 10 U of enzyme to digest 0.2 µg of PCR product at 37 °C for 3 h. Post-digestion, technical duplicates are pooled, and 2 µL of the resulting restricted DNA is combined with 15 µL of Hi-Di formamide for fragment analysis. This analysis is conducted on a SeqStudio device from (Thermo Fisher Scientific, Waltham, United States), with specific settings for injection time (40 s at 1200 Volts) and run time (1,440 s at 9000 Volts). The analysis is fine-tuned for fragments ranging from 20 to 500 base pairs (bp) with a bin width set at 5 bp, ensuring all samples are analyzed in a single run with normalization performed across the sum of signal intensities. The fragmentation patterns obtained from this analysis are meticulously examined using the R programming language, alongside the vegan community ecology package, allowing for in-depth analysis and calculations based on the peak areas of the fragments.

## Results and discussion

### Characterization of applied catalysts

The selection of rutile-TiO_2_ and g-C_3_N_4_ as basic catalysts for ARGs removal is underpinned by their advantageous characteristics, including low solubility, high-surface area, and tunable band gap. Furthermore, their versatile synthesis capabilities, diverse morphologies, and the flexibility for easy doping with various elements make them promising candidates for achieving enhanced compatibility and efficiency in specific contexts, particularly when considering the beneficial role of metal dopants in narrowing the band gap and generating highly oxidized species for efficient UV-visible light absorption.

**Fig. 1:**
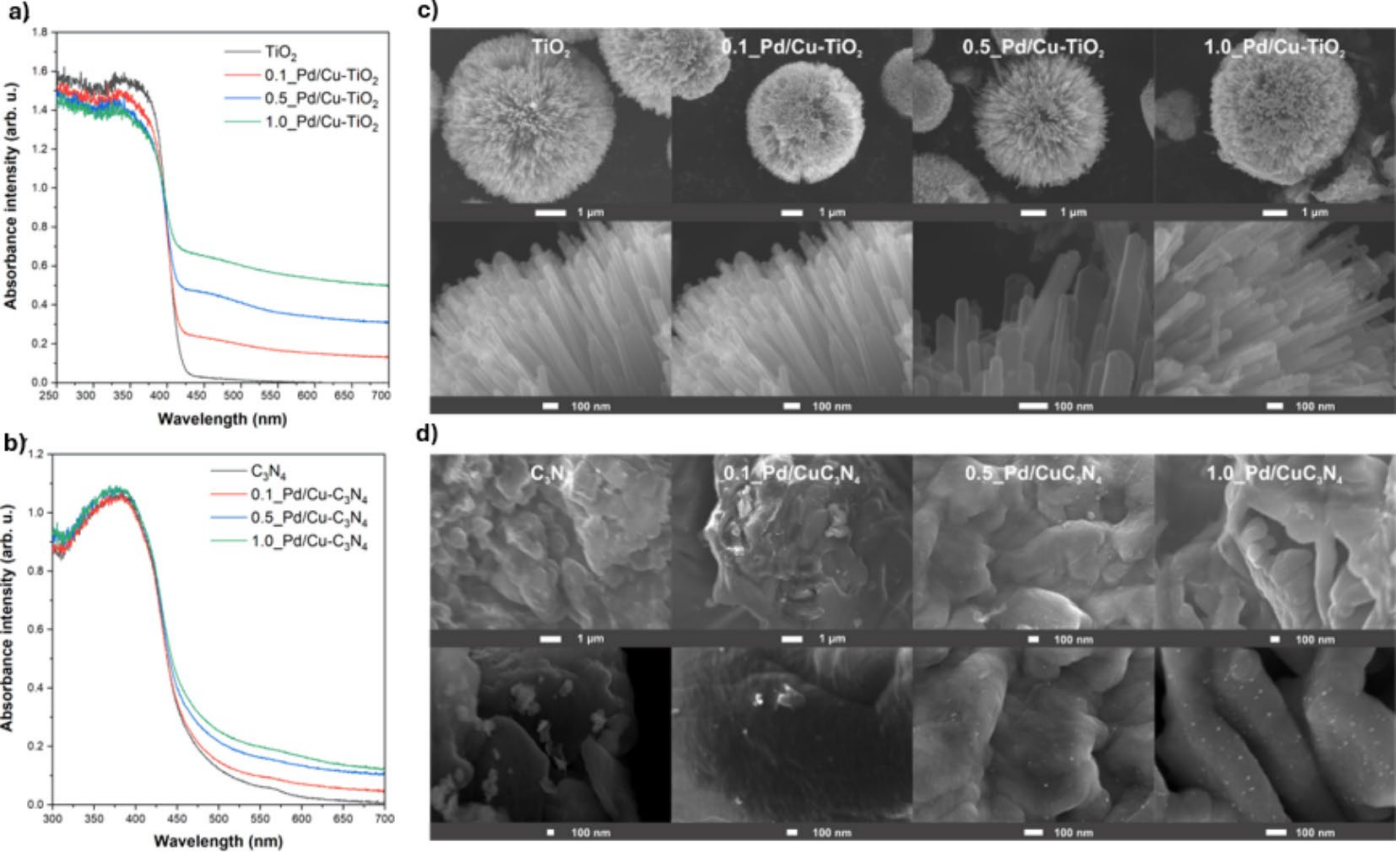
DRS UV-Vis spectra of (**a**) Pd/Cu-TiO_2_, (**b**) Pd/Cu-g-C_3_N_4_ photocatalysts and HR-SEM images of (**c**) Pd/Cu-TiO_2_ and (**d**) Pd/Cu-g-C_3_N_4_ photocatalysts.

Figure 1 shows diffuse reflectance spectra of synthesized bimetallic Pd/Cu of both rutile- TiO_2_ and g-C_3_N_4_ photocatalysts. In the cause of pristine and modified TiO_2_ samples the strongest photoabsorption was observed in the region below 420 nm due to rutile nature of TiO_2_ (Fig. 1a)^43^. On the other hand, pristine g-C_3_N_4_ and Pd/Cu- modified g-C_3_N_4_ exhibited the highest ability to absorb light in the range of 250–460 nm (Fig. 1b), which is in line with the band gap of C_3_N_4_ photocatalysts (2.7 eV)^44^. Additionally, modified catalysts exhibited an extended absorption in the visible range, above 420 nm and 480 nm for TiO_2_ and g-C_3_N_4_ based, respectively), which is related to the presence of Pd and Cu NPs on the surface of photocatalyst^45,46^. In both cases, the absorption intensity increased with increasing the amount of deposited bimetallic Pd/Cu NPs. Thus, introduction of Pd/Cu NPs significantly improved light absorption and could positively affect the photocatalytic properties.

Morphology of bimetallic modified Pd/Cu- TiO_2_ and g-C_3_N_4_ photocatalysts is presented in Fig. 1c, d, respectively. Pristine and modified g-C_3_N_4_ exhibited an irregular flake-like shapes. The size of this structures is at the range of 0.5–1.5 μm. TiO_2_ based photocatalysts shows the morphology of the spheres from which the rods grow (like sea urchins). Based on measurements of square side length, the individual rods showed the dimensions from 20 to 90 nm. While the diameter of whole spheres reached 6.1 ± 0.45 μm. In the case of modified samples, Pd/Cu spherical nanoparticles on the surface can be observed. As the amount of deposited nanoparticles increases, NPs become more visible.

### Inactivation via AOPs

As previously stated, conventional wastewater treatment is less efficacious in eliminating organic micropollutants (OMPs), ARB, and ARGs^47^. As a result, advanced treatment procedures are necessary to further remove the ARB and ARGs. This section endeavors to compare the outcomes of numerous AOPs methods (Table 2) in order to discover the optimum treatment process for destroying ARB and ARGs using targeted genes: *bla*_*TEM*_, *ermB*,* qnrS*,* tetM*. and 23 S rDNA gene is a taxonomic gene marker for one Gram-positive bacteria enterococci^47,48^. Apart from that, Rodriguez-Mozaz et al. (2015)^2^ has revealed that targeted ARGs, such as *bla*_*TEM*_ (resistance to beta-lactams), *ermB* (resistance to macrolides), *qnrS* (resistance to fluoroquinolones), and *tetM* (resistance to tetracyclines) were detected at the highest concentrations in hospital effluent and WWTP influent samples, posing a hazard to public health. Moreover, 16 S rDNA was chosen due to its widespread presence and stability, making it a reliable indicator for assessing overall eubacterial load reduction^49^. Under normal conditions, 16 S rDNA abundance in the WWTP effluent is around $\:1\times\:{10}^{8}\sim{10}^{9}$ cell equivalents/100mL^48^, and a comparable abundance of the gene 16 S rDNA was found in our analysis (2.17 $\:\times\:{10}^{9}$ cell equivalents 100 mL^−1^). While class 1 integrons, represented by *intl1*, are genetic mobile elements proposed as environmental indicators of acquired antibiotic resistance^50^. Hence, the presence of the universal class 1 integron-integrase gene *intl1* has been identified.

**Fig. 2:**
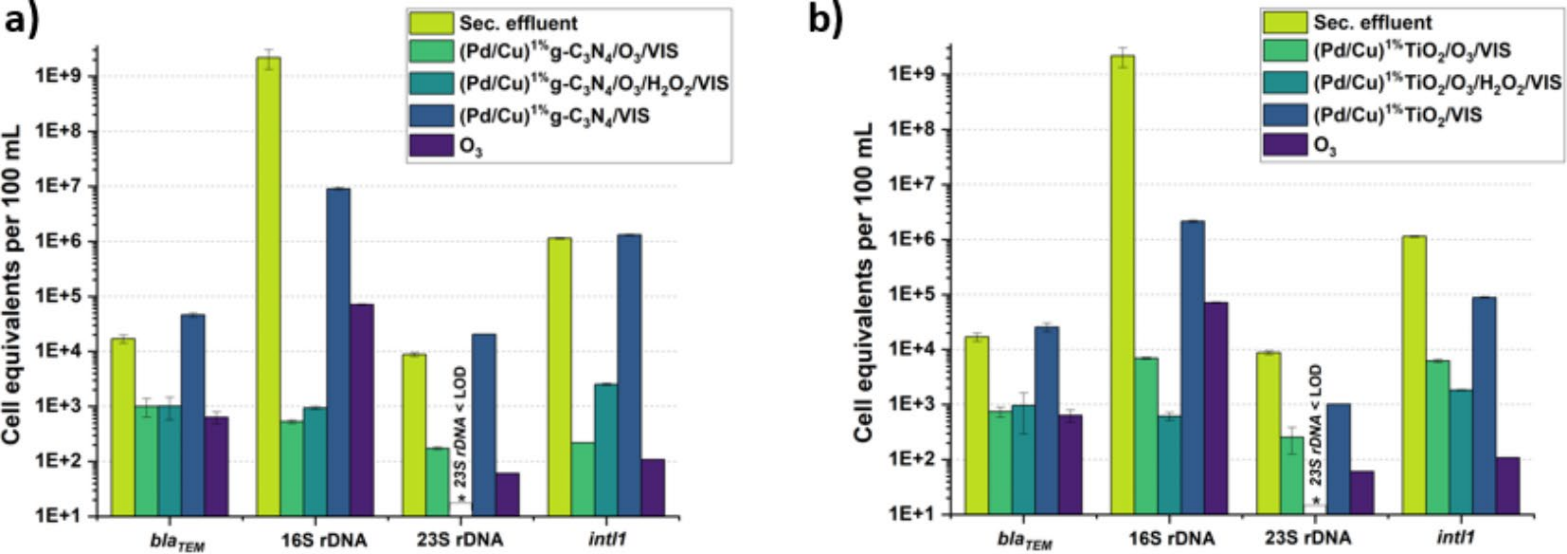
Influence of visible-light photocatalytic-based disinfection and processes on the absolute abundance of 16 S rDNA, *intl1*,* bla*_*TEM*_, 23 S rDNA: photocatalytic oxidation ((Pd/Cu)^1%^catalysts/VIS), photocatalytic ozonation ((Pd/Cu)^1%^catalysts/O_3_/VIS) and photocatalyst-assisted peroxone process ((Pd/Cu)^1%^catalysts/O_3_/H_2_O_2_/VIS *Blank column: below quantification/detection limit.

All experiments were conducted for 60 min as previous research indicated that longer ozone treatment (60 min) is more beneficial in reducing microorganisms to values below the limit of detection^47^. Based on the extant literature, ozonation appears to be an efficacious approach for attenuating micropollutants, ARB and ARGs^49,51^, as well as preventing bacterial regrowth, when used under optimal settings, such as a certain ozone dosage and exposure duration^49,52^. Given the varying susceptibility of different ARGs to ozonation, it becomes imperative to eradicate ARGs through tertiary treatment methods. Upon directing our attention solely towards the ozonation assays, as visually demonstrated in Fig. 2, the 16 S rDNA exhibited a decline of 4.5 logarithmic units, manifesting as the most vulnerable genetic material in contrast to other genes tested. Such a result corroborates the hierarchy of genetic vulnerability previously highlighted in the introduction, where the 16 S rDNA was observed to be more readily eliminated than ARGs^53^. Therefore, in the ozonation discussion part, the removal order is found to be: 16 S rDNA *> intl1 >* 23 S rDNA *> bla*_*TEM*_. It is worth noting that different bacterial species and related ARGs possess varying degrees of resistance or sensitivity to oxidative stress, which has already suggested by Alexander et al., 2016^51^. Additionally, investigations by Alexander et al. (2016)^51^ and Xu et al. (2016)^54^ reported limited removal of ARGs. In our study, some of the ARGs also appeared to be oxidation-tolerant, which could be due to the occurrence of HGT when DNA is damaged by ozone treatment. This suggests that while ozone may initially inactivate bacteria, the subsequent release of ARGs presents a significant challenge. The persistence of these ARGs, particularly due to HGT, complicates efforts to fully eliminate antibiotic resistance using oxidative treatments alone. Dodd (2012) discusses that ozone O_3_ is likely to inactivate ARB by first damaging the bacterial envelope before effectively degrading the ARGs^55^. This suggests that ARGs experience a delayed inactivation compared to ARB. As a result, there is a potential risk that ARGs may remain active, be repaired, or even be acquired by other ARB, thereby continuing the cycle of resistance even after the initial bacterial inactivation.

**Fig. 3:**
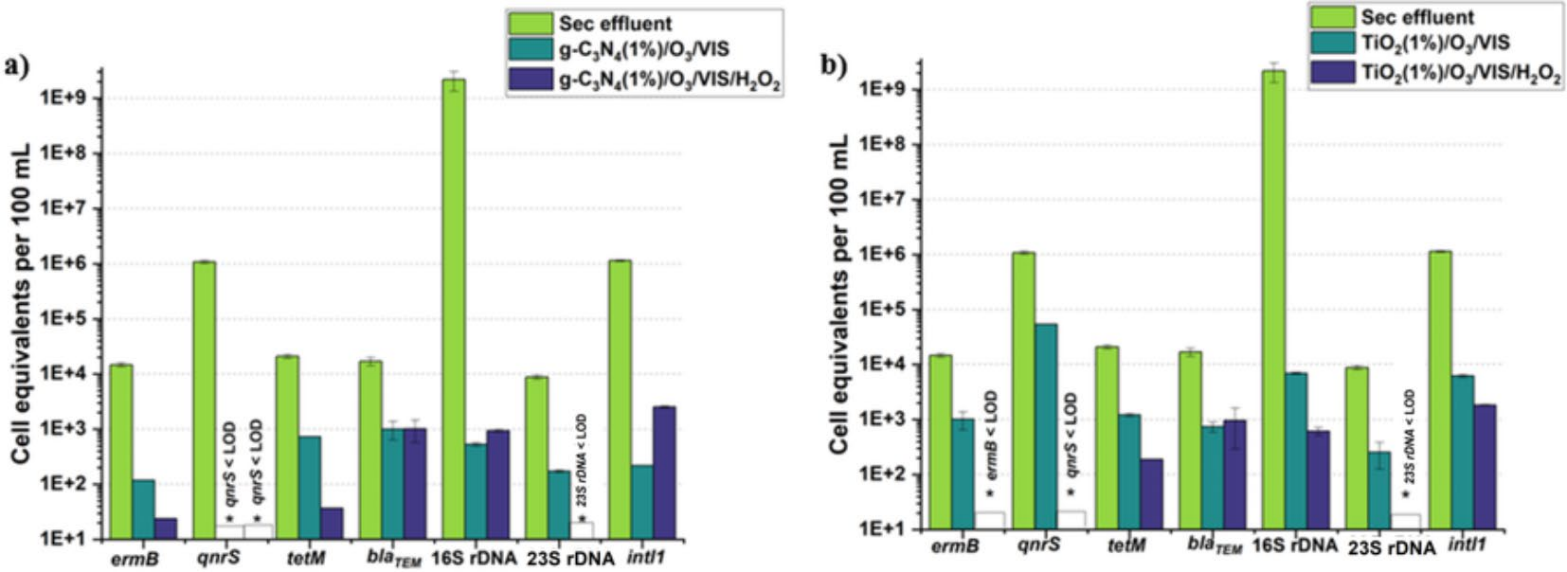
Comparison of the influence of applied AOPs’ ((Pd/Cu)^1%^catalysts/O_3_/VIS and (Pd/Cu)^1%^catalysts/O_3_/VIS/H_2_O_2_) on the absolute abundance of the genes *ermB*,* qnrS*,* tetM*,* bla*_*TEM*_, 16 S rDNA, 23 S rDNA, *intl1*1. *Blank column: below quantification/detection limit.

In our investigation, as depicted in Fig. 2 the abundance of 16 S rDNA reduced by 5.5 and 6.5 log units in rutile-TiO_2_-based and g-C_3_N_4_-based photocatalytic ozonation scenarios, respectively, and two treatment scenarios also resulted in 2.3 log units and 3.7 log units in *intl1* gene reduction, respectively. In the context of our investigation, it is noteworthy to observe that a reduction of 1.7 log units and 1.8 log units in 23 S rDNA was noted in rutile-TiO_2_-based and g-C_3_N_4_-based photocatalytic ozonation scenarios, respectively. Additionally, we observed a decrease of 1.4 log units and 1.2 log units in *bla*_*TEM*_ genes in rutile-TiO_2_-based and g-C_3_N_4_-based photocatalytic ozonation scenarios, respectively. Such reductions, although moderate in magnitude, suggest that these treatment modalities may exhibit a degree of efficiency in mitigating the dissemination of these resistance determinants in the aquatic milieu. However, In the absence of ozone intervention, with the exception of 16 S rDNA, the photocatalytic oxidation procedure yielded only marginal reduction of targeted genes. In other words, the abundance of *bla*_*TEM*_ gene exhibited no reduction in either situation. The genes 23 S rDNA and *intl1* indicated 1 log unit reduction by rutile-TiO_2_-based photocatalytic oxidation treatment, however, no reduction was observed in the circumstance treated by g-C_3_N_4_. Although 16 S rDNA gene was reduced by 3 log units and 2.3 log units in TiO_2_-based and g-C_3_N_4_-based treatment situations, respectively, it still indicated considerably less efficient than the other three advanced treatment methods (i.e. ozonation, photocatalytic ozonation, and photocatalyst-assisted peroxone process). Our finding led us to hypothesize that photocatalytic oxidation techniques are ineffective in removing ARGs. As a result of our work, treatment techniques including the addition of ozone have been demonstrated to effectively eliminate ARGs compared to conventional photocatalytic disinfection, which corresponds to the findings of previous studies^25,51^. When comparing the removal effectiveness of two bi-metallic catalysts following photocatalytic oxidation procedures (Fig. S2a, b), 1% CuPd TiO_2_ outperforms 1% CuPd g-C_3_N_4_ in photocatalytic oxidation treatment process; nevertheless, all targeted genes are more resilient to 1% CuPd TiO_2_ in photocatalytic ozonation trials. Several operational factors, including as catalyst loading, temperature, pH, and treated water characteristics, synthetic pathway of catalysts might result in differences in catalytic activity, which could be assessed by the rate of photo-introduced ^●^OH formation^56^. Furthermore, the actual transfer of electron and hole pairs, the recombination rate, and even the turbidity of the catalysts might result in varying degradation outcomes during the studies, therefore the explanation for this still requires further investigation. Furthermore, it is evident from Fig. 3 that the photocatalyst-assisted peroxone process do not significantly accelerate the efficiency as expected. However, the addition of H_2_O_2_ to photocatalytic ozonation treatment for both scenarios (TiO_2_-based and g-C_3_N_4_-based catalysts) reveal different mechanisms of ARGs inactivation, especially in terms of 16 S rDNA and *intl1*. Hence, additional exploration of this process is detailed in the following section.

### Photocatalyst-assisted peroxone process

**Fig. 4:**
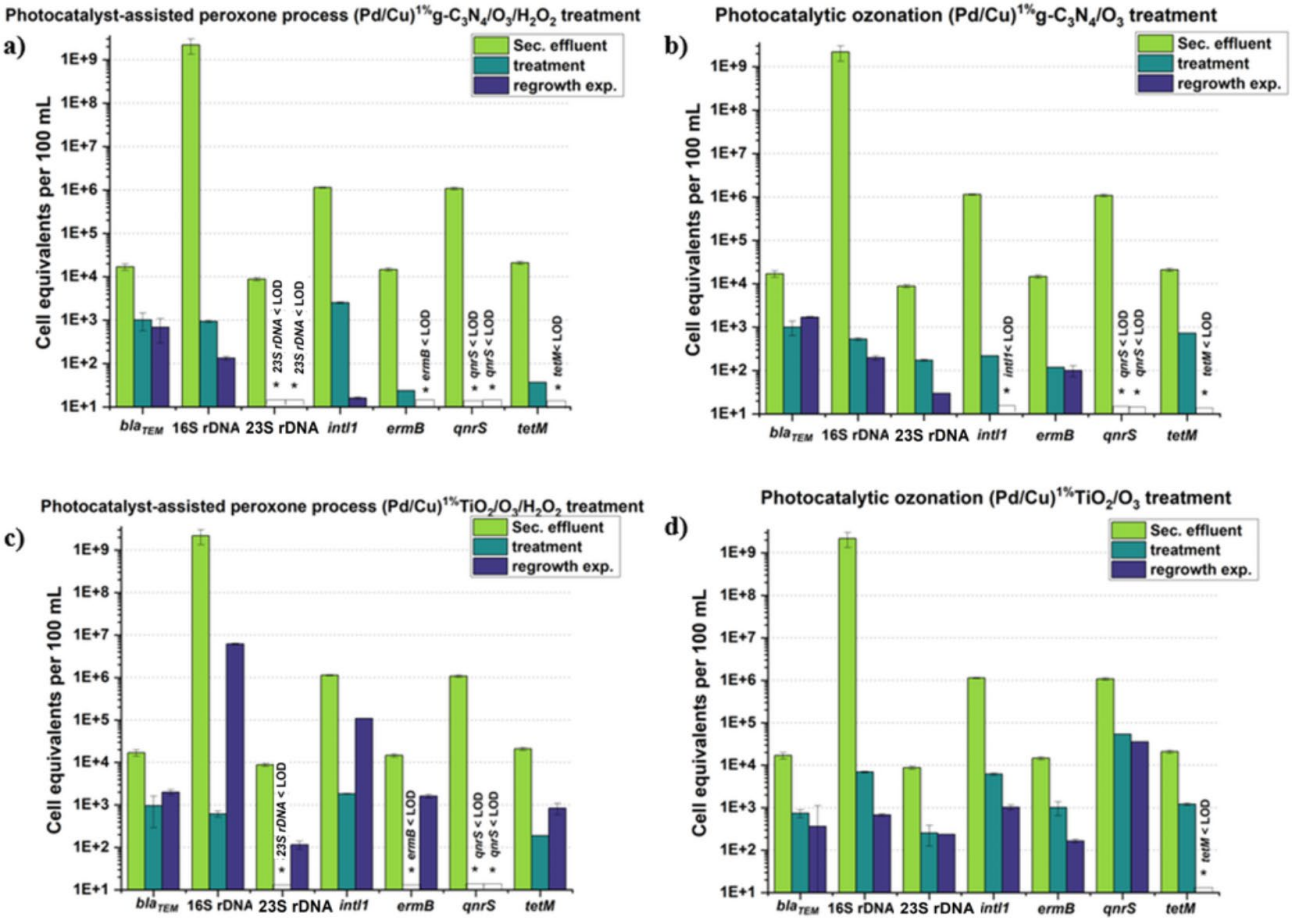
Comparison the performance of Cu/Pd^1%^(TiO_2_-based and g-C_3_N_4_-based) photocatalytic ozonation (with/without H_2_O_2_) in suppressing *bla*_*TEM*_, 16 S rDNA, 23 S rDNA, *intl1*,* ermB*,* qnrS*,* tetM* regrowth. *Blank column: below quantification/detection limit.

As it was mentioned above, the difference in inactivation efficiency caused by (Pd/Cu)^1%^catalysts/O_3_/H_2_O_2_/VIS has to be explored. As such, Ternes et al. (2003)^57^ revealed that ozone-based AOPs incorporating H_2_O_2_ boosted oxidation efficiency in contrast to single ozonation, since the peroxone process (O_3_/H_2_O_2_) was associated with improving experiment outcomes by increasing ozone degradation and ^●^OH generation rate^58^. Our findings revealed that, in both catalytic scenarios (Fig. 3, from left graph to right graph) with the presence of hydrogen peroxide, 16 S rDNA was reduced by up to 6.6 log and 6.8 log units, while *intl1* was reduced by up to 2.6 log units and 2.8 log unit, respectively. The abundance of *bla*_*TEM*_ was found to undergo a reduction of 1.3 log units in both catalytic scenarios. The elimination effectiveness of 16 S rDNA and *bla*_*TEM*_ were compared. The removal effectiveness of *bla*_*TEM*_ against 16 S rDNA gene reduction suggested that *bla*_*TEM*_ had a lower removal efficiency. The *bla*_*TEM*_ gene appears to be the most resistant among the ARGs examined, demonstrating substantial resilience to treatment, a finding that aligns with the study published by Gmurek et al., 2023^25^. This discovery bears paramount importance, as the treatment procedures entail the disruption of cellular membranes through the involvement of ROS^59^. Consequently, the ubiquitous 16 S rDNA gene, which serves as a housekeeping gene, was expected to be more susceptible to elimination than ARGs. Apart from that, the 23 S rDNA gene’s qPCR results shown in Fig. 4a, b are both close to the limit of quantification (LOQ), indicating that the gene has been fully eliminated by the treatment procedure. The genomes with a high GC content are resistant to ozonation treatments^60^. Ozone-sensitive bacteria are defined as enterococci having a guanine-cytosine (GC) content less than 40%, which explains why the 23 S rDNA gene appeared to be readily eliminated^61,62^. Other researchers^63^ examined at the possibility of the TiO_2_-P25 photocatalytic approach in removing genes (16 S, *intl1*,* bla*_*TEM*_), and reported that the treatment technique lowered all of the studied genes marginally. However, when H_2_O_2_ was added to the process, the deletion of targeted genes increased, indicating that our observations are valid. In general, the extra H_2_O_2_ in the solar-driven AOPs would enhance the removal efficiencies of ARB, ARGs, and pharmaceutical compounds, as observed by previous studies^35,49,64^. The genes of *ermB*, *qnrS*, and 23 S rDNA were completely removed employing TiO_2_-based photocatalyst-assisted peroxone process. Furthermore, *ermB* and *tetM* genes were only partly eliminated by g-C_3_N_4_-based photocatalyst-assisted peroxone process, whereas *qnrS* gene was completely deleted. Ferro et al. (2016)^65^ observed that after 240 min of UV/H_2_O_2_ treatment, the *bla*_*TEM*_ gene climbed to $\:3.7\times\:{10}^{3}$ copies/ml in total DNA, with no difference in the *qnrS* gene between the beginning ($\:5.1\times\:{10}^{4}$copies/mL) and final sample ($\:4.3\times\:{10}^{4}$ copies/mL). It is evident that when compared to the other genes, the *qnrS* gene is the most sensitive for reactions involving H_2_O_2_. Despite the fact that this phenomenon implied that photocatalytic treatment, even with spiking H_2_O_2_, was inefficient in eliminating ARGs^65^. As a result of the transformation and elimination of plasmid DNA by ozone and photocatalytic treatment procedures^66^, O_3_ coupled with photocatalytic treatment plays a crucial role in eradicating ARGs in our research. According to the discussion, as shown in Fig. 3, following ranking of treatment efficiency can be made: (Pd/Cu)^1%^catalysts/O_3_/H_2_O_2_/VIS > (Pd/Cu)^1%^catalysts/O_3_/VIS > O_3_ > (Pd/Cu)^1%^catalysts/VIS. The effectiveness of photocatalyst-assisted peroxone has been corroborated in various studies. Among the various combinations of AOPs and photocatalysis (particularly those involving TiO_2_), the TiO_2_/UV/O_3_/H_2_O_2_ (peroxone) process has emerged as the most effective treatment for wastewater. This method is also considered the most cost-efficient based on economic evaluations, making it an optimal choice for large-scale applications^39^.

As previously elucidated, titanium dioxide demonstrates commendable efficacy in mitigating ARGs within the context of a photocatalytic oxidation milieu. Nevertheless, its performance in ARG removal is notably inferior when juxtaposed with the discernibly superior outcomes achieved by g-C_3_N_4_ in a photocatalytic ozonation scenario. After the incorporation of H_2_O_2_ in the photocatalytic ozonation system, as illustrated in Fig. 4, genes *qnrS* and 23 S rDNA display significant susceptibility in both catalytic scenarios. Conversely, for genes *ermB*, *intl1*, 16 S rDNA, the presence of TiO_2_ not only enhances treatment efficiency compared to the situation without H_2_O_2_ but also demonstrates superior improvement compared to the g-C_3_N_4_ scenario. In relation to the gene *tetM*, g-C_3_N_4_-based photocatalyst-assisted peroxone process, proves to be more effective in its removal. Conversely, the advanced treatment appears to have no discernible effect on the gene *bla*_*TEM*_ compared to the scenario in the absence of H_2_O_2_. These findings highlight the intricate relationship between diverse photocatalytic materials and treatment conditions when tackling antibiotic resistance genes. They underscore the importance of adopting customized approaches that are tailored to the specific genes in question and align with the objectives of the treatment.

### Bacterial regrowth

Within this section, all treated wastewater underwent disinfection through AOPs following which each sample was split in half and stored under ambient temperature in a light-free environment for a period of either 24–72 h, contingent on the specific treatment processes that had been applied. The investigation focused on the proliferation of bacteria following the aforementioned storage durations of 24 and 72 h. The reason for this was that bacteria might persist in viability and subsequently regenerate even after the removal of stress. According to our findings, bimetallic rutile-TiO_2_ and g-C_3_N_4_ with 1% doped Cu/Pt seemed to have the highest suppressive performance with ozone application, while 1% doped Cu/Pd was not the optimal loading for eliminating targeted genes (23 S rDNA and *intl1*) as shown in Fig. S3. We aimed to explore photocatalyst-assisted peroxone process, photocatalytic ozonation, and photocatalytic oxidation in inhibiting bacterial regrowth after storage using 1% doped Cu/Pd catalysts because of the intriguing behavior.

According to the Fig. 4a–d, after 3-days of storage, none of the targeted genes were observed to be close to their pre-treatment levels, demonstrating that photocatalytic ozonation may greatly suppress bacterial growth in both H_2_O_2_ and non-H_2_O_2_ circumstances. The gene 23 S rDNA and *qnrS* were completely inactivated following photocatalyst-assisted peroxone process (Fig. 4a), even after 3-days of storage, no regeneration was observed with g-C_3_N_4_-based catalyst. With the exception of the 23 S rDNA and *qnrS* genes, which were eliminated in a circumstance where there was no bacterial regrowth after adding H_2_O_2_, there was also no evidence of bacterial growth in the *ermB* and *tetM* genes. However, when the absence of H_2_O_2_ was examined (Fig. 4b), in comparison to the presence of H_2_O_2_, genes 23 S rDNA and *ermB* remain detectable even after a 24-hour period of dark incubation. When the effectiveness of TiO_2_-based treatment methods with and without H_2_O_2_ was compared, the photocatalyst-assisted peroxone process demonstrated better ARG elimination efficiency but poorer bacterial regrowth inhibition. As illustrated in Fig. 4c, the advanced treatment exhibited remarkable efficacy in completely eradicating the gene 23 S rDNA and *ermB*. Nevertheless, upon subjecting the samples to a subsequent period of dark storage, a noteworthy resurgence of both genetic markers was observed. In addition to the mentioned genes, other targeted genes also exhibited substantial regrowth; however, the gene copy numbers did not surpass the original data despite this resurgence. Based on the data presented in Fig. 4d, it is evident that, except for the gene *tetM*, all the targeted genes remain detectable even after 24 h of dark storage. However, the gene copy number were not sufficient to reach the levels observed in the original data. The same intriguing discovery was reported by Moreira et al. (2018)^63^, who investigated how solar-driven AOPs affect targeted genes (16 S, *intl1*,* bla*_*TEM*_), revealing that TiO_2_-P25/H_2_O_2_ had the best results in eradicating ARGs, but also suggested a major bacterial regeneration phenomenon. It is conceivable that when both TiO_2_ and H_2_O_2_ are being used in photocatalytic disinfection, undesirable bacterial proliferation could be observed. As a result, although TiO_2_-based photocatalyst-assisted peroxone process showed poor regeneration performance in ARGs, different ARGs showed variable regrowth performance. Based on our results, it is speculated that residual O_3_ and H_2_O_2_ could hinder the reactivation of microbes during storage. Nevertheless, it appears that the efficiency of the peroxone process, when aided by photocatalysts, in preventing regrowth is variable and largely depends on the specific catalysts used. Furthermore, when comparing the bacterial inhibitory ability of two catalysts, g-C_3_N_4_-based catalysts displayed superior potential in suppressing bacterial regeneration, suggesting that the hypothesis might be also based on various catalysts and targeted ARGs.

**Fig. 5:**
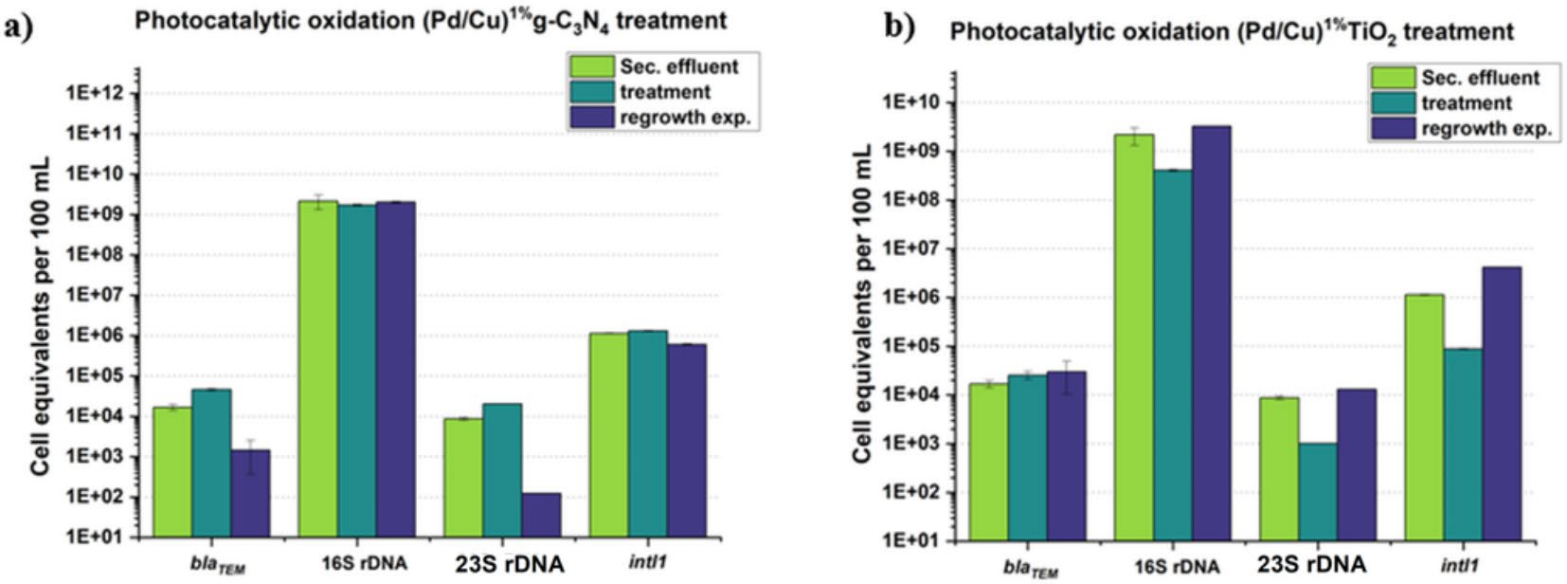
Comparison the performance of Cu/Pd^1%^ (TiO_2_-based and g-C_3_N_4_-based) photocatalytic oxidation in suppressing bacterial regrowth. *Blank column: below quantification/detection limit.

All targeted genes exhibited significant microbial reactivation after 3-days storage (Fig. 5a, b), according to the results of photocatalytic oxidation, and the abundance of 16 S *rDNA* was similar to or even higher than before treatment in both catalytic scenarios, which corresponds to the research of Moreira et al. (2018)^63^. Furthermore, when two bimetallic catalysts were examined, it was discovered that the g-C_3_N_4_-based photocatalytic oxidation was more effective at inhibiting bacterial proliferation, which was also validated in the previous statement. Based on our findings, it seems feasible to combine UV and chlorination to minimize bacterial recurrence due to photocatalytic oxidation’s insufficient bacterial inhibitory ability.

### PCR-DGGE and t-RFLP analyses

**Fig. 6:**
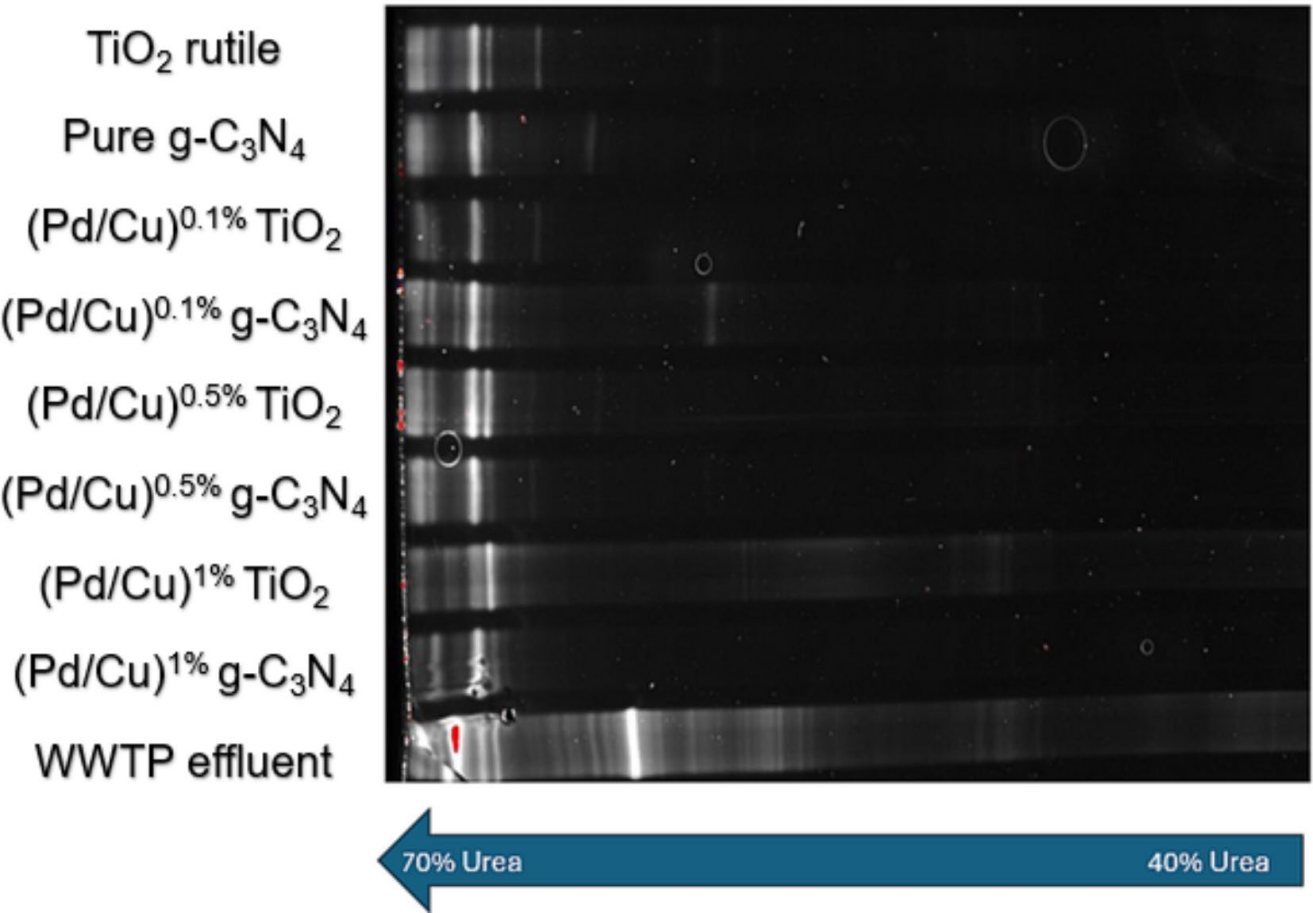
Nine discrete population analyses were independently conducted employing the PCR-DGGE methodology. Specifically, the eubacterial primers GC27F and 517R were utilized to amplify the V1–V2 variable regions of the 16 S rDNA.

The study aimed to assess the distinct impact of various doping catalysts on the bacterial diversity within the wastewater community. The PCR-DGGE methodology was employed to investigate the bacterial population across different water samples. Through the PCR reaction, millions of copies of DNA strands were generated from the template DNA, as outlined by Bharagava et al. (2018)^67^. DGGE, a molecular fingerprinting technique, was utilized to separate DNA strands through acrylamide gel electrophoresis containing a urea gradient (40–70%) based on sequence variations, as described by Chandel et al. (2022)^68^. As depicted in Fig. 6, all DNA strands were effectively separated and visualized as distinct bands. In the column representing WWTP effluent, a diverse array of distinct bands was evident, substantiating the high level of bacterial contamination in the raw wastewater passing the conventional wastewater treatment at the municipal WWTP. The DNA band pattern mirrors a high diversity with different Guanin/Cytosin (G/C) contents. Here, the G/C contents of the amplified PCR product of the 16 S rDNA impacts the migration rate along the urea gradient: the higher the G/C contents the higher the migration due to the stronger hydrogen bonds between the G/C bases^69^. However, following photocatalytic ozonation, only a sparse number of bands were discernible at the lower portion of the gels, indicating the effectiveness of the treatment method in eliminating microbial contaminants. It is noteworthy that there was not a substantial discrepancy observed among the different scenarios involving various doping catalysts. In accordance with the findings presented by Alexander et al. (2016)^51^, it is established that bacteria possessing a high GC content tend to manifest heightened stability, maintaining their double-stranded configuration until reaching to the conditions of elevated denaturant concentrations. This structural robustness is attributed to the greater number of hydrogen bonds existing between GC nucleotide pairs as mentioned before. From this premise, we posit that bacterial strains characterized by an elevated GC content may exhibit an augmented resilience to advanced treatment technologies, such as ozonation and photocatalytic ozonation. Up to now, we can´t predict that these ozone robust and selected bacteria might have a direct link to ARB or certain ARGs, this need to be analyzed in future.

As discussed earlier, in all photocatalytic ozonation scenarios, the bands of bacteria significantly decreased (Fig. 6). Interestingly, a shift in band occurrence, as well as relatively brighter or more intense bands, was observed for different catalysts. Particularly highlighting the significant impact of core TiO_2_ and g-C_3_N_4_ based catalysts on distinct inactivation mechanisms. Additionally, slight differences were noticed in various Cu/Pd loadings for both TiO_2_ and g-C_3_N_4_-based catalysts, indicating that bacteria may be slightly more susceptible to g-C_3_N_4_-based catalytic ozonation than TiO_2_-based processes.

In addition to the DGGE technique, t-RFLP analysis was employed to examine the biodiversity of each treatment. The results of this analysis, along with Bray-Curtis and Shannon metrics, are presented in the Supplementary Information (Tables S3 and S4). The Bray-Curtis index, ranging from 0 to 1, is a metric used to gauge biodiversity variations between samples subjected to AOPs and the untreated control. A coefficient of 0 suggests identical species compositions, while 1 implies no shared species. Meanwhile, the Shannon coefficient measures biodiversity within a single microbiome, with higher values indicating greater diversity. Analysis of the results reveals a consistent range of Bray-Curtis indices (0.79 to 0.85) for samples treated solely with O_3_. However, when both O_3_ and H_2_O_2_ were applied, the indices surged to around 0.9, indicating a pronounced increase in biodiversity differences compared to the untreated sample.

In the context of the bacterial community (Table S4), there was no statistically significant difference between g-C_3_N_4_ and TiO_2_-based catalysts in photocatalytic ozonation with 0.5% (Pd/Cu) bimetallic catalysts. However, the 0.1% Pd/Cu modified catalysts showed lower biodiversity for TiO_2_ compared to g-C_3_N_4_. This trend persisted with 1% Pd/Cu bimetallic modification, indicating a lower Shannon index for g-C_3_N_4_ than TiO_2_ catalysts (2.19 and 2.95, respectively), aligning with DGGE results. Further insights from the Shannon index demonstrate that biodiversity coefficients for samples treated with the combination of O_3_ and H_2_O_2_ were notably lower than those for samples subjected to other treatment methods. Photocatalyst-assisted peroxone process caused higher bacterial diversity in g-C_3_N_4_-based catalysts than TiO_2_-based (Shannon index 2.14 and 1.47, respectively). This suggests that, concerning biodiversity within a single population, samples treated with both O_3_ and H_2_O_2_ exhibit reduced diversity compared to those treated with alternative methods.

## Conclusion

This study highlights the critical importance of advanced wastewater treatment methods in effectively removing ARB and ARGs, demonstrating their capability to mitigate and prevent the spread of these contaminants. Various AOPs were evaluated, with photocatalytic ozonation, particularly when augmented with H₂O₂, proving to be highly effective. The findings also reveal variability in tolerance among different bacterial species and their associated ARGs. In terms of catalyst performance, g-C₃N₄ demonstrated superior efficiency over TiO₂ in removing ARB and ARGs and in preventing bacterial regeneration. The choice of catalyst significantly affects disinfection outcomes, with g-C₃N₄ proving more effective in this context. Overall, a significant decrease in microbial community diversity was observed, influenced by catalyst type and loading. The Photocatalyst-Assisted Peroxone Process notably impacted the Alpha-Diversity of treated samples, emphasizing its potential for advancing treatment protocols to address environmental challenges related to FPB and ARGs while safeguarding aquatic ecosystems.

## Electronic supplementary material

Below is the link to the electronic supplementary material.

Supplementary Material 1

## Data Availability

The datasets used and/or analysed during the current study available from the corresponding author on reasonable request.
