# Supplementary material for: The role of TiO2 and gC3N4 bimetallic catalysts in boosting antibiotic resistance gene removal through photocatalyst assisted peroxone process
Source: Sci Rep. 2024 Oct 2;14:22897. doi: 10.1038/s41598-024-74147-4 (PMC11447026; doi:10.1038/s41598-024-74147-4)
Supplement: Supplementary file 1 — Supplementary Material 1 [file 41598_2024_74147_MOESM1_ESM.pdf]

Supplementary Information

The Role of TiO_2 and gC_3N_4 Bimetallic Catalysts in Boosting Antibiotic Resistance Gene Removal through Photocatalyst Assisted Peroxone Process

Cong Xiaoyu[1], Paweł Mazierski[4], Magdalena Miodyńska[4], Adriana Zaleska-Medynska[4], Harald Horn [3,5], Thomas Schwartz [1], Marta Gmurek [1,2,3]

[1]Karlsruhe Institute of Technology (KIT), Institute of Functional Interfaces (IFG), Microbiology/Molecular Biology Department, Hermann von Helmholtz Platz 1, 76344 Eggenstein-Leopoldshafen, Germany.

[2]Department of Molecular Engineering, Faculty of Process and Environmental Engineering, Lodz University of Technology, 90-924 Lodz, Poland

[3]Karlsruhe Institute of Technology, Engler-Bunte-Institut, Water Chemistry and Water Technology, 76131 Karlsruhe, Germany

[4]Department of Environmental Technology, Faculty of Chemistry, University of Gdansk, 80-308, Gdansk, Poland

[5]DVGW German Technical and Scientific Association for Gas and Water Research Laboratories, Water Chemistry and Water Technology, 76131 Karlsruhe, Germany

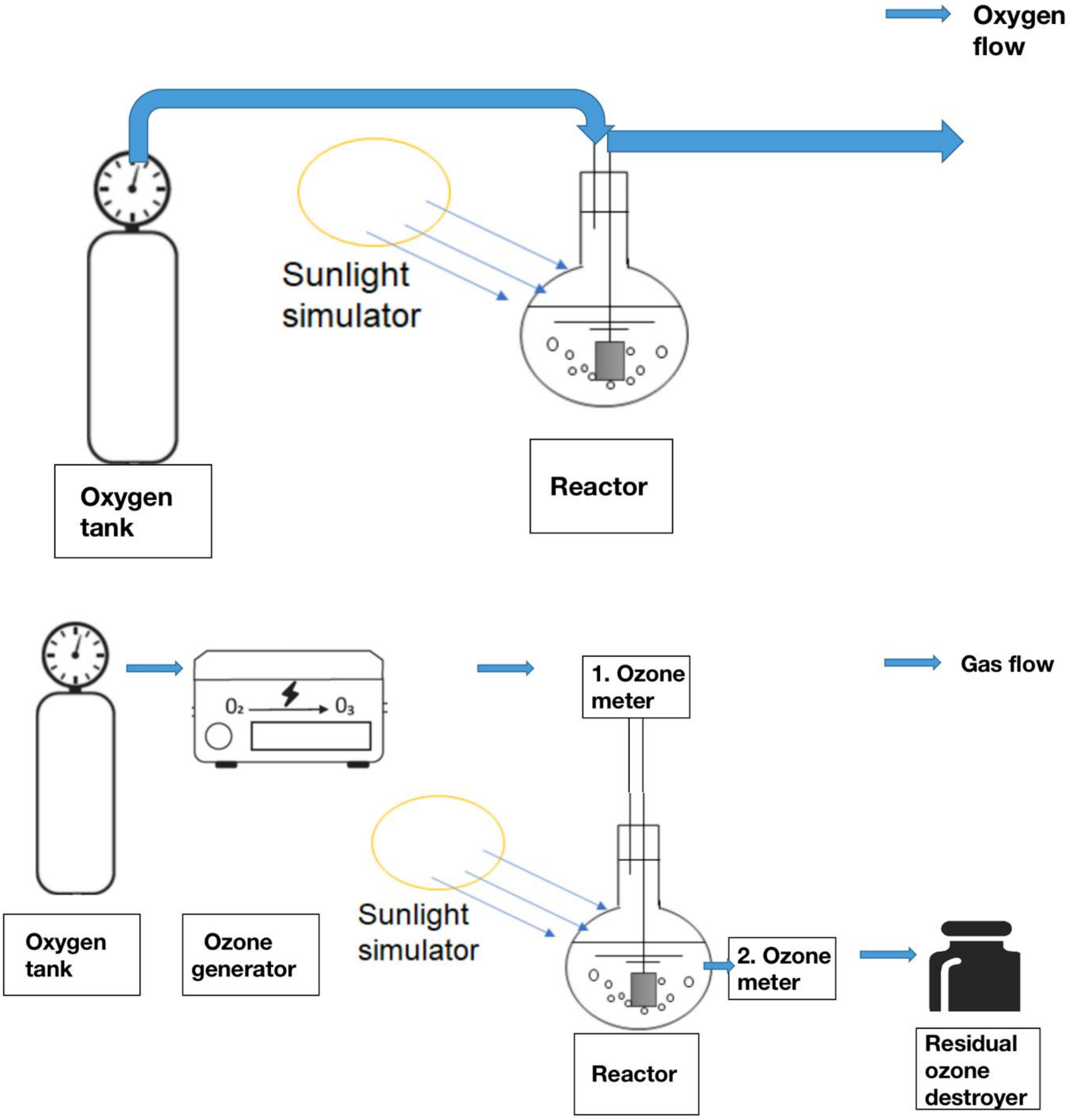

Figure S1.: Diagram of the photocatalytic ozonation unit's process flow

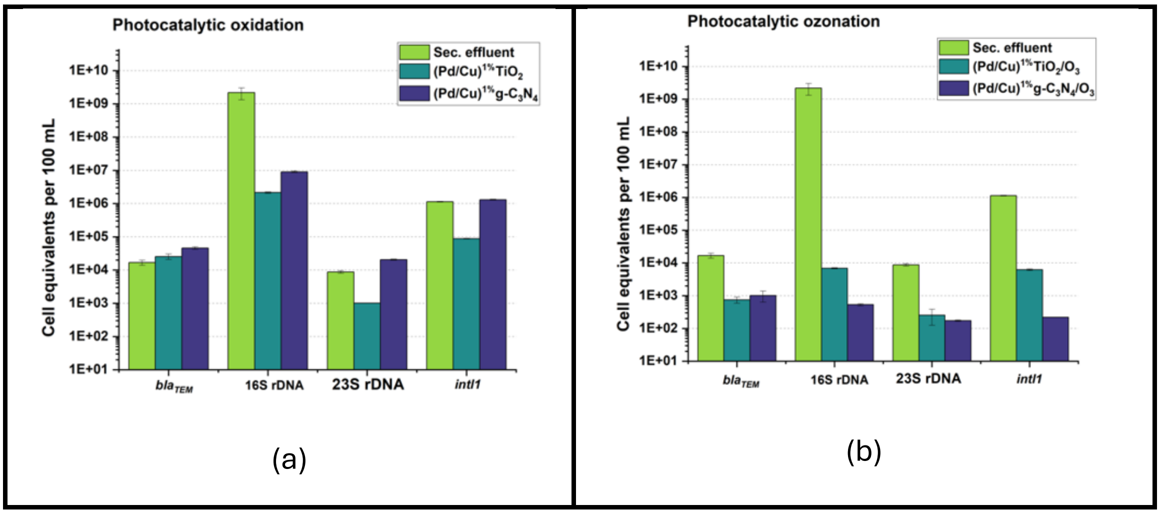

Figure S2: Comparison of the removal rates of ARGs (16S rDNA, *bla*$_{TEM}$, *intl1*, and 23S rRNA) by applied catalysts (Pd/Cu)1%TiO_2 and (Pd/Cu)1% g-C_3N_4) in two photocatalytic scenarios

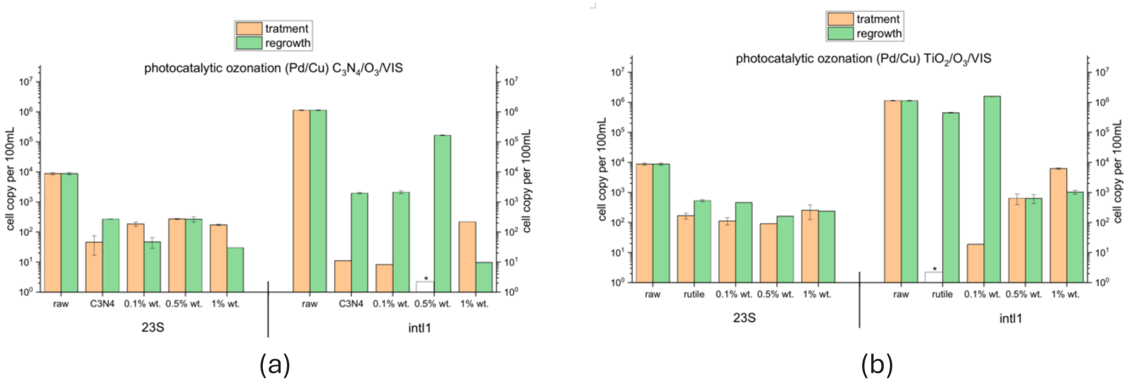

Figure S3: Assessing the effectiveness of both catalysts with diverse metallic dopants in inhibiting bacterial regrowth and eliminating genes 23S rDNA and intl1.

Table S1: Characteristics of secondary effluent from Karlsruhe WWTP

**Parameter Unit**	**Secondary effluent from WWTP**
DOC	10.5 ± 0.4 mgL^{-1}
COD	31 ± 4 mgL^{-1}
pH	7.7 ± 0.5
$NH_4^+ - N$	11.4 ± 0.5 mgL^{-1}
$PO_4^{3-} - P$	$1.3+0.0$ mgL^{-1}

Table S2: Primer List for the Detection of Opportunistic Bacteria, Antibiotic Resistance Genes, and Amplification of 16S rDNA Fragments for Population Analysis.

Target	Primer sequence	Equation of the calibration curve	Amplicon size	efficiency	R^2	LOD	Control strain	reference
**Facultative pathogenic bacteria**								
Enterococcus spp.	Fwd: AGAAATTCCAAACGAACTTG<br>Rev: CAGTGCTCTACCTCCATCATT	F(x)= -3,585x+35,283	93 bp	90,1 %	1,000	65	*E. faecium* DSM20477	(Frahm & Obst, 2003)
16S rDNA	Fwd: TCCTACGGGAGGCAGCAGT<br>Rev: ATTACCGCGGCTGCTGG	F(x)= -3,405x+35,350	195bp	95,5%	0,997		*E. coli* pNORM	
**Antibiotic resistance genes**								
*erm*B	Fwd: TGAATCGAGACTTGAGTGTGCAA<br>Rev: GGATTCTACAAGCGTACCTT	F(x)=-3,328x+35,901	71 bp	100 %	1,000	16	*S. hyointestinalis* DSM20770	(Alexander et al., 2015)
*Int*I1	Fwd: GCCTTGATGTTACCCGAGAG<br>Rev: GATCGGTCGAATGCGTGT	F(x)= -3,472x+34,720	196 bp	94,1 %	1,000	126	*E. coli* pNORM	(Rocha et al., 2020)
*tet*M	Fwd:GGTTTCTCTTGGATACTTAAATCAATC<br>Rev: CCAACCATAAATCCTTGTTCRC	F(x)=-3,424x+38,747	88 bp	95,9 %	0,998	4	*E. coli* DH5α	(Peak et al., 2007)
*bla*TEM	Fwd: TTCCTGTTTTTGCTCACCCAG<br>Rev: CTCAAGGATCTTACCGCTGTTG	F(x)=-3,303x+38,559	112 bp	100,8 %	0,999	80	*E. coli* pNORM	(Rocha et al., 2020)
qnrS	Fwd: F-5′-ATGGAAACCTACAATCATACATATCGG-3′<br>Rev: R-5′-TTAGTCAGGATAAACAACAATACCC-3′)	F(x)=-3,448x+35,589	657 bp	95%	0,999		E. coli DH5α	(Zhao et al., 2021)
**PCR-DGGE and t-RFLP**								
Ribosomal DNA	GC27F: AGAGTTTGATCCTGGCTCAG<br>Fam-27F:AGAGTTTGATCCTGGCTCAG FAM labeled<br>517R: ATTACCGCGGCTGCTGG		509bp				*Eubacteria*	(Muyzer et al., 1993)

Table S3: Bray–Curtis coefficient of all the AOPs treatment.

	**Control**	**TiO_2(rutile) $+O_3$**	**gC_3N_4(pure) $+O_3$**	**$(Pd/Cu)^{0.1\%}TiO_2$ $+O_3$**	**$(Pd/Cu)^{0.1\%}$ gC_3N_4 $+O_3$**	**$(Pd/Cu)^{0.5\%}TiO_2$ $+O_3$**	**$(Pd/Cu)^{0.5\%}$ gC_3N_4 $+O_3$**	**$(Pd/Cu)^{1\%}TiO_2$ $+O_3$**	**$(Pd/Cu)^{1\%}$ gC_3N_4 $+O_3$**	**$(Pd/Cu)^{1\%}TiO_2$ $+O_3+H_2O_2$**	**$(Pd/Cu)^{1\%}$ $gC_3N_4+O_3+H_2O_2$**
**Control**	0										
**TiO_2(rutile) $+O_3$**	0,81	0									
**gC_3N_4(pure) $+O_3$**	0,82	0,37	0								
**$(Pd/Cu)^{0.1\%}TiO_2$ $+O_3$**	0,79	0,28	0,39	0							
**$(Pd/Cu)^{0.1\%}$ gC_3N_4 $+O_3$**	0,81	0,23	0,28	0,22	0						
**$(Pd/Cu)^{0.5\%}TiO_2$ $+O_3$**	0,79	0,15	0,38	0,24	0,21	0					
**$(Pd/Cu)^{0.5\%}$ gC_3N_4 $+O_3$**	0,82	0,18	0,31	0,34	0,21	0,22	0				
**$(Pd/Cu)^{1\%}TiO_2$ $+O_3$**	0,85	0,72	0,70	0,72	0,68	0,66	0,70	0			
**$(Pd/Cu)^{1\%}$ gC_3N_4 $+O_3$**	0,71	0,79	0,80	0,76	0,78	0,78	0,80	0,86	0		
**$(Pd/Cu)^{1\%}TiO_2$ $+O_3+H_2O_2$**	0,92	0,76	0,76	0,78	0,76	0,76	0,75	0,76	0,90	0	
**$(Pd/Cu)^{1\%}$ $gC_3N_4+O_3+H_2O_2$**	0,91	0,79	0,78	0,78	0,75	0,74	0,76	0,66	0,91	0,22	0

Table S4: Shannon coefficient of all the AOPs treatment.

Treatment	Shannon coefficient	
	TiO_2(rutile)	gC_3N_4
Pristine Catalysts+O_3	1.95	2.30
$(Pd/Cu)^{0.1\%}$+O_3	2.05	2.32
$(Pd/Cu)^{0.5\%}$ +O_3	2.22	2.21
$(Pd/Cu)^{1\%}$+O_3	2.95	2.19
$(Pd/Cu)^{1\%}$+O_3+H_2O_2	1.47	2.14

References

Alexander, J., Bollmann, A., Seitz, W., & Schwartz, T. (2015). Microbiological characterization of aquatic microbiomes targeting taxonomical marker genes and antibiotic resistance genes of opportunistic bacteria. *Science of the Total Environment*, *512*, 316–325. https://doi.org/10.1016/j.scitotenv.2015.01.046

Frahm, E., & Obst, U. (2003). Application of the fluorogenic probe technique (TaqMan PCR) to the detection of Enterococcus spp. and Escherichia coli in water samples. *Journal of Microbiological Methods*, *52*(1), 123–131. https://doi.org/10.1016/S0167-7012(02)00150-1

Muyzer, G., De Waal, E. C., & Uitterlinden, A. G. (1993). Profiling of complex microbial populations by denaturing gradient gel electrophoresis analysis of polymerase chain reaction-amplified genes coding for 16S rRNA. *Applied and Environmental Microbiology*, *59*(3), 695. https://doi.org/10.1128/AEM.59.3.695-700.1993

Peak, N., Knapp, C. W., Yang, R. K., Hanfelt, M. M., Smith, M. S., Aga, D. S., & Graham, D. W. (2007). Abundance of six tetracycline resistance genes in wastewater lagoons at cattle feedlots with different antibiotic use strategies. *Environmental Microbiology*, *9*(1), 143–151. https://doi.org/10.1111/J.1462-2920.2006.01123.X

Rocha, J., Cacace, D., Kampouris, I., Guilloteau, H., Jäger, T., Marano, R. B. M., Karaolia, P., Manaia, C. M., Merlin, C., Fatta-Kassinos, D., Cytryn, E., Berendonk, T. U., & Schwartz, T. (2020). Inter-laboratory calibration of quantitative analyses of antibiotic resistance genes. *Journal of Environmental Chemical Engineering*, *8*(1), 102214. https://doi.org/10.1016/J.JECE.2018.02.022

Zhao, Y., Cao, Z., Cui, L., Hu, T., Guo, K., Zhang, F., Wang, X., Peng, Z., Liu, Q., & Dai, M. (2021). Enrofloxacin Promotes Plasmid-Mediated Conjugation Transfer of Fluoroquinolone-Resistance Gene qnrS. *Frontiers in Microbiology*, *12*. https://doi.org/10.3389/FMICB.2021.773664
